# 4-Chloro-*N*-(3-chloro­phen­yl)benzene­sulfonamide

**DOI:** 10.1107/S1600536811011366

**Published:** 2011-03-31

**Authors:** K. Shakuntala, Sabine Foro, B. Thimme Gowda

**Affiliations:** a Department of Chemistry, Mangalore University, Mangalagangotri 574 199, Mangalore, India; b Institute of Materials Science, Darmstadt University of Technology, Petersenstrasse 23, D-64287, Darmstadt, Germany

## Abstract

In the crystal of the title compound, C_12_H_9_Cl_2_NO_2_S, the mol­ecule is twisted at the S atom with a C—SO_2_—NH—C torsion angle of −58.4 (3)°. Furthermore, the N—H bond in this segment is *anti* to the *meta*-chloro group. The dihedral angle between the aromatic rings is 77.1 (1)°. The crystal structure features inversion-related dimers linked by N—H⋯O hydrogen bonds.

## Related literature

For our study on the effect of substituents on the structures of *N*-(ar­yl)aryl­sulfonamides, see: Gowda *et al.* (2005 ▶); Shakuntala *et al.* (2011 ▶). For the effect of substituents on the oxidative strengths of *N*-chloro,*N*-aryl­sulfonamides, see: Gowda & Shetty (2004 ▶) and for the effect of substituents on the NQR spectra of *N*-(ar­yl)-amides, see: Gowda *et al.* (2000 ▶).
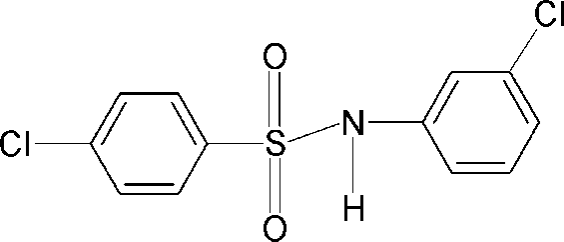


## Experimental

### 

#### Crystal data

C_12_H_9_Cl_2_NO_2_S
*M* *_r_* = 302.16
Monoclinic, 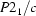

*a* = 9.378 (2) Å
*b* = 13.478 (3) Å
*c* = 10.251 (2) Å
β = 90.48°
*V* = 1295.6 (5) Å^3^
*Z* = 4
Mo *K*α radiation
μ = 0.65 mm^−1^
*T* = 293 K
0.48 × 0.44 × 0.44 mm

#### Data collection

Oxford Diffraction Xcalibur diffractometer with Sapphire CCD detector
Absorption correction: multi-scan (*CrysAlis RED*; Oxford Diffraction, 2009 ▶) *T*_min_ = 0.745, *T*_max_ = 0.762
4232 measured reflections
2115 independent reflections
1572 reflections with *I* > 2σ(*I*)
*R*_int_ = 0.014

#### Refinement

*R*[*F* ^2^ > 2σ(*F* ^2^)] = 0.047
*wR*(*F* ^2^) = 0.129
*S* = 1.08
2115 reflections
166 parameters
1 restraint
H atoms treated by a mixture of independent and constrained refinement
Δρ_max_ = 0.54 e Å^−3^
Δρ_min_ = −0.49 e Å^−3^

### 

Data collection: *CrysAlis CCD* (Oxford Diffraction, 2009 ▶); cell refinement: *CrysAlis RED* (Oxford Diffraction, 2009 ▶); data reduction: *CrysAlis RED*; program(s) used to solve structure: *SHELXS97* (Sheldrick, 2008 ▶); program(s) used to refine structure: *SHELXL97* (Sheldrick, 2008 ▶); molecular graphics: *PLATON* (Spek, 2009 ▶); software used to prepare material for publication: *SHELXL97*.

## Supplementary Material

Crystal structure: contains datablocks I, global. DOI: 10.1107/S1600536811011366/ds2101sup1.cif

Structure factors: contains datablocks I. DOI: 10.1107/S1600536811011366/ds2101Isup2.hkl

Additional supplementary materials:  crystallographic information; 3D view; checkCIF report

## Figures and Tables

**Table 1:** Hydrogen-bond geometry (Å, °)

*D*—H⋯*A*	*D*—H	H⋯*A*	*D*⋯*A*	*D*—H⋯*A*
N1—H1*N*⋯O1^i^	0.85 (2)	2.09 (2)	2.939 (4)	176 (4)

Symmetry code: (i) 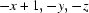.

**Fig. 1.**
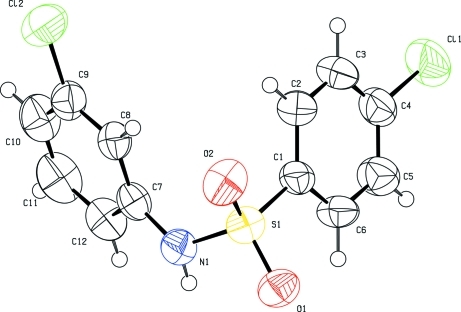
Molecular structure of (I), showing the atom labelling scheme and displacement ellipsoids are drawn at the 50% probability level.

**Fig. 2.**
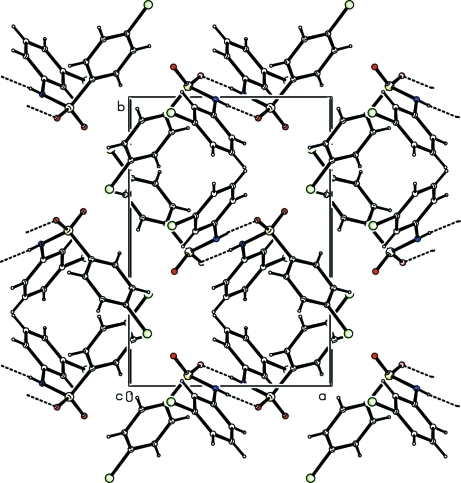
Molecular packing of (I) with hydrogen bonding shown as dashed lines.

## supplementary crystallographic information

### Comment

The amide and sulfonamide moieties are important constituents of many biologically important compounds. As a part of studying the substituent effects on the structures and other aspects of this class of compounds (Gowda *et al.*, 2000, 2004, 2005; Shakuntala *et al.*, 2011), in the present work, the crystal structure of 4-chloro-*N*-(3-chlorophenyl)benzenesulfonamide (I) has been determined (Fig. 1). The molecule is bent at the S atom with the C—SO_2_—NH—C torsion angle of -58.4 (3)°, compared to the value of -56.7 (2)° in 4-chloro-*N*-(2,3-dichlorophenyl)-benzenesulfonamide (II) (Shakuntala *et al.*, 2011). The N—H bond and the *meta*- chloro group in the anilino benzene ring are *anti* to each other.

The sulfonyl and the anilino benzene rings in (I) are tilted relative to each other by 77.1 (1)°, compared to the value of 56.5 (1)° in (II).

Inermolecular N—H···O(S) hydrogen bonding interactions generates inversion related dimers which are further packed via van der Waals interactions in the crystal structure (Fig.2).

### Experimental

The solution of chlorobenzene (10 ml) in chloroform (40 ml) was added dropwise with chlorosulfonic acid (25 ml) at 0 ° C. After the initial evolution of hydrogen chloride subsided, the reaction mixture was brought to room temperature and poured into crushed ice in a beaker. The chloroform layer was separated, washed with cold water and allowed to evaporate slowly. The residual 4-chlorobenzenesulfonylchloride was treated with 3-chloroaniline in the stoichiometric ratio and boiled for ten minutes. The reaction mixture was then cooled to room temperature and added to ice cold water (100 ml). The resultant 4-chloro-*N*-(3-chlorophenyl)-benzenesulfonamide was filtered under suction and washed thoroughly with cold water. It was then recrystallized to constant melting point from dilute ethanol. The compound was characterized by FT-IR and NMR spectra.

Prism like colorless single crystals used in X-ray diffraction studies were grown in ethanolic solution by slow evaporation at room temperature.

### Refinement

The H atom of the NH group was located in a difference map and later restrained to the distance N—H = 0.86 (2) Å. The other H atoms were positioned with idealized geometry using a riding model with C—H = 0.93 Å A l l H atoms were refined with isotropic displacement parameters (set to 1.2 times of the *U*_eq_ of the parent atom). The weak diffraction of the crystal resulted in low theta value. However, the refinement went well

### Crystal data

**Table:**

C_12_H_9_Cl_2_NO_2_S	*F*(000) = 616
*M**_r_* = 302.16	*D*_x_ = 1.549 Mg m^−^^3^
Monoclinic, *P*2_1_/*c*	Mo *K*α radiation, λ = 0.71073 Å
Hall symbol: -P 2ybc	Cell parameters from 959 reflections
*a* = 9.378 (2) Å	θ = 2.9–27.8°
*b* = 13.478 (3) Å	µ = 0.65 mm^−^^1^
*c* = 10.251 (2) Å	*T* = 293 K
β = 90.48°	Prism, colourless
*V* = 1295.6 (5) Å^3^	0.48 × 0.44 × 0.44 mm
*Z* = 4	

### Data collection

**Table:**

Oxford Diffraction Xcalibur diffractometer with Sapphire CCD detector	2115 independent reflections
Radiation source: fine-focus sealed tube	1572 reflections with *I* > 2σ(*I*)
graphite	*R*_int_ = 0.014
Rotation method data acquisition using ω and phi scans.	θ_max_ = 24.7°, θ_min_ = 3.0°
Absorption correction: multi-scan (*CrysAlis RED*; Oxford Diffraction, 2009)	*h* = −11→10
*T*_min_ = 0.745, *T*_max_ = 0.762	*k* = −15→15
4232 measured reflections	*l* = −11→11

### Refinement

**Table:**

Refinement on *F*^2^	Primary atom site location: structure-invariant direct methods
Least-squares matrix: full	Secondary atom site location: difference Fourier map
*R*[*F*^2^ > 2σ(*F*^2^)] = 0.047	Hydrogen site location: inferred from neighbouring sites
*wR*(*F*^2^) = 0.129	H atoms treated by a mixture of independent and constrained refinement
*S* = 1.08	*w* = 1/[σ^2^(*F*_o_^2^) + (0.047*P*)^2^ + 1.4846*P*] where *P* = (*F*_o_^2^ + 2*F*_c_^2^)/3
2115 reflections	(Δ/σ)_max_ = 0.006
166 parameters	Δρ_max_ = 0.54 e Å^−^^3^
1 restraint	Δρ_min_ = −0.49 e Å^−^^3^

### Special details

**Table:**

Experimental. Empirical absorption correction using spherical harmonics, implemented in SCALE3 ABSPACK scaling algorithm.
Geometry. All e.s.d.'s (except the e.s.d. in the dihedral angle between two l.s. planes) are estimated using the full covariance matrix. The cell e.s.d.'s are taken into account individually in the estimation of e.s.d.'s in distances, angles and torsion angles; correlations between e.s.d.'s in cell parameters are only used when they are defined by crystal symmetry. An approximate (isotropic) treatment of cell e.s.d.'s is used for estimating e.s.d.'s involving l.s. planes.
Refinement. Refinement of *F*^2^ against ALL reflections. The weighted *R*-factor *wR* and goodness of fit *S* are based on *F*^2^, conventional *R*-factors *R* are based on *F*, with *F* set to zero for negative *F*^2^. The threshold expression of *F*^2^ > σ(*F*^2^) is used only for calculating *R*-factors(gt) *etc*. and is not relevant to the choice of reflections for refinement. *R*-factors based on *F*^2^ are statistically about twice as large as those based on *F*, and *R*- factors based on ALL data will be even larger.

### Fractional atomic coordinates and isotropic or equivalent isotropic displacement parameters (Å^2^)

**Table:**

	*x*	*y*	*z*	*U*_iso_*/*U*_eq_	
C1	0.1825 (3)	−0.0615 (3)	0.0799 (3)	0.0508 (9)	
C2	0.0654 (4)	−0.0812 (4)	0.1546 (4)	0.0729 (12)	
H2	0.0440	−0.0410	0.2255	0.087*	
C3	−0.0207 (4)	−0.1612 (4)	0.1236 (5)	0.0825 (14)	
H3	−0.0999	−0.1755	0.1743	0.099*	
C4	0.0106 (4)	−0.2191 (3)	0.0188 (4)	0.0679 (11)	
C5	0.1265 (4)	−0.1999 (3)	−0.0564 (4)	0.0689 (11)	
H5	0.1468	−0.2399	−0.1278	0.083*	
C6	0.2127 (4)	−0.1211 (3)	−0.0256 (4)	0.0596 (10)	
H6	0.2923	−0.1078	−0.0763	0.072*	
C7	0.4235 (4)	−0.0627 (2)	0.3131 (4)	0.0505 (8)	
C8	0.3302 (4)	−0.0357 (3)	0.4113 (3)	0.0516 (8)	
H8	0.2676	0.0172	0.4000	0.062*	
C9	0.3323 (4)	−0.0887 (3)	0.5251 (4)	0.0584 (9)	
C10	0.4236 (5)	−0.1658 (3)	0.5465 (5)	0.0743 (12)	
H10	0.4237	−0.2000	0.6254	0.089*	
C11	0.5151 (5)	−0.1916 (3)	0.4491 (5)	0.0784 (13)	
H11	0.5784	−0.2438	0.4620	0.094*	
C12	0.5152 (4)	−0.1415 (3)	0.3323 (4)	0.0631 (10)	
H12	0.5769	−0.1607	0.2664	0.076*	
O1	0.3569 (3)	0.07335 (19)	−0.0026 (2)	0.0640 (7)	
O2	0.2238 (3)	0.1039 (2)	0.1997 (3)	0.0728 (8)	
N1	0.4324 (3)	−0.0091 (2)	0.1956 (3)	0.0562 (8)	
H1N	0.491 (3)	−0.030 (3)	0.139 (3)	0.067*	
Cl1	−0.09656 (14)	−0.31895 (11)	−0.01890 (13)	0.1052 (5)	
Cl2	0.21587 (13)	−0.05483 (10)	0.64754 (11)	0.0900 (4)	
S1	0.29685 (10)	0.03746 (7)	0.11668 (9)	0.0552 (3)	

### Atomic displacement parameters (Å^2^)

**Table:**

	*U*^11^	*U*^22^	*U*^33^	*U*^12^	*U*^13^	*U*^23^
C1	0.0405 (17)	0.060 (2)	0.052 (2)	0.0058 (15)	−0.0001 (15)	0.0121 (18)
C2	0.052 (2)	0.108 (3)	0.059 (2)	0.000 (2)	0.0080 (19)	−0.003 (2)
C3	0.047 (2)	0.124 (4)	0.077 (3)	−0.018 (2)	0.006 (2)	0.021 (3)
C4	0.058 (2)	0.077 (3)	0.069 (3)	−0.016 (2)	−0.0094 (19)	0.016 (2)
C5	0.067 (2)	0.066 (3)	0.074 (3)	−0.012 (2)	0.005 (2)	−0.002 (2)
C6	0.055 (2)	0.062 (2)	0.062 (2)	−0.0103 (18)	0.0159 (17)	0.003 (2)
C7	0.0478 (18)	0.0433 (18)	0.060 (2)	−0.0041 (15)	−0.0076 (16)	−0.0038 (17)
C8	0.0511 (19)	0.0509 (19)	0.053 (2)	0.0036 (16)	−0.0068 (16)	−0.0009 (17)
C9	0.061 (2)	0.059 (2)	0.055 (2)	−0.0050 (18)	−0.0063 (18)	0.0001 (19)
C10	0.077 (3)	0.060 (3)	0.086 (3)	−0.002 (2)	−0.018 (2)	0.014 (2)
C11	0.075 (3)	0.049 (2)	0.112 (4)	0.012 (2)	−0.018 (3)	0.005 (2)
C12	0.059 (2)	0.051 (2)	0.079 (3)	0.0060 (18)	−0.003 (2)	−0.013 (2)
O1	0.0741 (17)	0.0581 (15)	0.0598 (16)	−0.0056 (13)	0.0076 (13)	0.0121 (12)
O2	0.090 (2)	0.0604 (16)	0.0680 (17)	0.0245 (15)	0.0041 (14)	−0.0037 (14)
N1	0.0513 (18)	0.0630 (19)	0.054 (2)	0.0002 (15)	0.0007 (14)	−0.0036 (16)
Cl1	0.0940 (9)	0.1148 (11)	0.1066 (10)	−0.0559 (8)	−0.0191 (7)	0.0267 (8)
Cl2	0.0911 (8)	0.1135 (10)	0.0657 (7)	0.0044 (7)	0.0125 (6)	0.0095 (7)
S1	0.0592 (5)	0.0493 (5)	0.0572 (6)	0.0048 (4)	0.0031 (4)	0.0025 (4)

### Geometric parameters (Å, °)

**Table:**

C1—C2	1.370 (5)	C7—N1	1.407 (5)
C1—C6	1.379 (5)	C8—C9	1.367 (5)
C1—S1	1.751 (4)	C8—H8	0.9300
C2—C3	1.383 (6)	C9—C10	1.364 (5)
C2—H2	0.9300	C9—Cl2	1.732 (4)
C3—C4	1.362 (6)	C10—C11	1.367 (6)
C3—H3	0.9300	C10—H10	0.9300
C4—C5	1.362 (5)	C11—C12	1.374 (6)
C4—Cl1	1.722 (4)	C11—H11	0.9300
C5—C6	1.370 (5)	C12—H12	0.9300
C5—H5	0.9300	O1—S1	1.434 (2)
C6—H6	0.9300	O2—S1	1.416 (3)
C7—C12	1.380 (5)	N1—S1	1.627 (3)
C7—C8	1.387 (5)	N1—H1N	0.846 (18)
			
C2—C1—C6	119.8 (4)	C7—C8—H8	120.7
C2—C1—S1	121.3 (3)	C10—C9—C8	122.7 (4)
C6—C1—S1	118.9 (3)	C10—C9—Cl2	118.9 (3)
C1—C2—C3	119.5 (4)	C8—C9—Cl2	118.4 (3)
C1—C2—H2	120.3	C9—C10—C11	118.2 (4)
C3—C2—H2	120.3	C9—C10—H10	120.9
C4—C3—C2	119.9 (4)	C11—C10—H10	120.9
C4—C3—H3	120.0	C10—C11—C12	121.1 (4)
C2—C3—H3	120.0	C10—C11—H11	119.4
C3—C4—C5	121.1 (4)	C12—C11—H11	119.4
C3—C4—Cl1	119.7 (3)	C11—C12—C7	119.8 (4)
C5—C4—Cl1	119.2 (4)	C11—C12—H12	120.1
C4—C5—C6	119.3 (4)	C7—C12—H12	120.1
C4—C5—H5	120.4	C7—N1—S1	124.9 (2)
C6—C5—H5	120.4	C7—N1—H1N	117 (3)
C5—C6—C1	120.5 (3)	S1—N1—H1N	107 (3)
C5—C6—H6	119.8	O2—S1—O1	119.65 (17)
C1—C6—H6	119.8	O2—S1—N1	108.95 (17)
C12—C7—C8	119.6 (4)	O1—S1—N1	104.09 (16)
C12—C7—N1	118.4 (3)	O2—S1—C1	108.23 (17)
C8—C7—N1	121.9 (3)	O1—S1—C1	108.50 (16)
C9—C8—C7	118.5 (3)	N1—S1—C1	106.73 (16)
C9—C8—H8	120.7		
			
C6—C1—C2—C3	0.4 (6)	C9—C10—C11—C12	−0.2 (6)
S1—C1—C2—C3	−178.8 (3)	C10—C11—C12—C7	1.2 (6)
C1—C2—C3—C4	−0.6 (6)	C8—C7—C12—C11	−1.1 (5)
C2—C3—C4—C5	0.4 (7)	N1—C7—C12—C11	176.1 (3)
C2—C3—C4—Cl1	179.7 (3)	C12—C7—N1—S1	143.4 (3)
C3—C4—C5—C6	0.0 (6)	C8—C7—N1—S1	−39.5 (5)
Cl1—C4—C5—C6	−179.3 (3)	C7—N1—S1—O2	58.2 (3)
C4—C5—C6—C1	−0.3 (6)	C7—N1—S1—O1	−173.1 (3)
C2—C1—C6—C5	0.0 (6)	C7—N1—S1—C1	−58.4 (3)
S1—C1—C6—C5	179.3 (3)	C2—C1—S1—O2	−18.4 (4)
C12—C7—C8—C9	0.1 (5)	C6—C1—S1—O2	162.4 (3)
N1—C7—C8—C9	−177.0 (3)	C2—C1—S1—O1	−149.6 (3)
C7—C8—C9—C10	1.0 (6)	C6—C1—S1—O1	31.2 (3)
C7—C8—C9—Cl2	179.9 (3)	C2—C1—S1—N1	98.8 (3)
C8—C9—C10—C11	−0.9 (6)	C6—C1—S1—N1	−80.5 (3)
Cl2—C9—C10—C11	−179.9 (3)		

### Hydrogen-bond geometry (Å, °)

**Table:**

*D*—H···*A*	*D*—H	H···*A*	*D*···*A*	*D*—H···*A*
N1—H1N···O1^i^	0.85 (2)	2.09 (2)	2.939 (4)	176 (4)

Symmetry codes: (i) −*x*+1, −*y*, −*z*.
